# Improved emotion regulation following a trauma-informed CBT-based intervention associates with reduced risk for recidivism in justice-involved emerging adults

**DOI:** 10.3389/fpsyt.2022.951429

**Published:** 2022-10-05

**Authors:** Nathalie M. Dumornay, Katherine E. Finegold, Anisha Chablani, Lili Elkins, Sotun Krouch, Molly Baldwin, Soo Jeong Youn, Luana Marques, Kerry J. Ressler, Alisha Moreland-Capuia

**Affiliations:** ^1^Division of Depression & Anxiety Disorders, McLean Hospital, Belmont, MA, United States; ^2^Institute of Child Development, University of Minnesota, Minneapolis, MN, United States; ^3^School and Child Clinical Psychology, University of Toronto, Toronto, ON, Canada; ^4^Roca, Inc., Chelsea, MA, United States; ^5^Department of Psychiatry, Massachusetts General Hospital, Boston, MA, United States; ^6^Department of Psychiatry, Harvard Medical School, Boston, MA, United States

**Keywords:** trauma-informed, juvenile justice, system change, PTSD, institutional racism, brain development, adverse childhood experiences (ACE's), healing

## Abstract

**Objective:**

Male youth who have been involved in the juvenile legal system have disproportionate rates of trauma and violence exposure. Many justice-involved youth have untreated mental illness, with an estimated 66% of young men who are incarcerated meeting criteria for at least one mental health disorder, including posttraumatic stress disorder (PTSD), depression, and substance abuse. While Cognitive Behavioral Therapy (CBT) approaches are considered among effective evidence-based treatments for addressing and treating behavioral and emotional difficulties, male youth with a history of incarceration and youth who are at risk for (re)incarceration, violence, emotion dysregulation, and trauma face significant barriers in accessing these services.

**Methods:**

Roca, Inc. (Roca), an internationally recognized organization moving the needle on urban violence by working relentlessly with young people at the center of violence in Massachusetts and Maryland, employs a trauma-informed CBT-based skills curriculum and approach in their intervention model, to improve youths' educational, employment, parenting, and life skills opportunities, while decreasing risk for recidivism, addressing trauma and increasing skills for emotion regulation. The aim of this analysis was to assess the effectiveness of Roca's trauma-informed CBT skills curriculum on youths' emotional and behavioral outcomes. We analyzed data from over 300 participating emerging adult men from four sites in Massachusetts and one site in Baltimore, Maryland who had at least three series of data collection across multiple skills-based sessions.

**Results:**

We found improvements in outcomes in overall mean scores related to decreased distress about employment and education, as are expected with standard intervention approaches for justice-involved youth. Participants who show improvement in emotion regulation across engagement (approximately half the cohort), were found to have significant improvements in distress related to relationship and family functioning and self-care, and decreased substance use, along with other outcomes compared to those participants with less improvement in emotion regulation. Furthermore, improvement in different aspects of emotion regulation were associated with improved relationships, life distress, substance use, and improved prosocial thinking.

**Conclusions:**

Together, these data suggest that adding mental health support and skills training, such as with trauma-informed CBT models, to programs for justice-involved youth may lead to significant improvements in functioning, quality of life, and mental health outcomes.

## Introduction

Male youth who have been involved in the juvenile legal system have disproportionate rates of trauma and violence exposure (1–3). Many justice-involved youths have untreated mental illness, with ~66% of young men who are incarcerated meeting criteria for at least one mental health disorder, including depressive disorders, posttraumatic stress disorder (PTSD), and substance use disorder (1, 4). Moreover, it is estimated that 90% of justice-involved youth have experienced at least one traumatic event (5). Despite knowledge of the associations between trauma, mental illness, and justice involvement, the historical approach to addressing youth violence and recidivism risk has noticeably lacked a nuanced consideration of the role that mental health and trauma-exposure plays in justice involvement (6). Addressing youth's underlying mental health issues could potentially impact their risk for (re)incarceration, since mental health problems and emotion regulation difficulties are cited as among significant risk factors for recidivism (7, 8). Furthermore, while previous literature provides supporting evidence that the more effective violence intervention programs place an emphasis on social, economic, developmental, and behavioral elements, access and referral to services is largely unavailable to youth involved in the juvenile legal system (9).

To address this barrier, Roca, a behavioral health intervention organization in Massachusetts and Maryland, added a Cognitive Behavioral Theory (CBT)-based curriculum and approach to their intervention model. The individuals that Roca serves are living at the center of urban violence and are determined to be at high risk for (re)incarceration, have experienced significant levels of trauma, have a history of engagement in violence, and/or have low educational attainment and limited or no work history (10, 11). Roca employs a CBT approach to engage emerging adult men, aged 17–24, who otherwise would not be eligible or able to participate in other youth driven services due to being high-risk for violence and (re)incarceration. There is compelling evidence that “meeting individuals where they are” by acknowledging and addressing behavioral and socioeconomic conditions can improve population health and is effective in disrupting the cycle of violence (12, 13). A large proportion of young people involved with the juvenile legal system are deemed transition age youth (i.e., 16–25 years of age) (14). This is a particularly vulnerable subgroup in the justice system, as this age group has the highest rates of mental health problems (15). Zajac et al. (14) highlighted that while several validated treatments target delinquency in justice-involved adolescents**, **there are fewer treatments established to address the unique developmental needs and mental health challenges in transitional age, justice involved youth. Transition aged youth, like those Roca serves, are at a heightened risk for the onset of major mental health disorders (16). Moreover, transition age youth face significant life changes, including educational and vocational transitions, greater independence from family, and changes in social influences/networks (14, 17, 18).

Prior literature shows a clear association between trauma-exposure, mental illness, and justice involvement (7, 19, 20). Systematic reviews indicate that interventions targeting mental health, such as multisystemic therapy and family focused therapy, are linked to outcomes such as decreased emotional and behavioral problems, decreased association with deviant peers, and decreased recidivism in antisocial youth referred from various systems, including the juvenile legal system (15, 21–24). Further, systematic reviews have indicated that certain trauma-informed interventions, most notably trauma-focused CBT, are linked with positive outcomes (e.g., pre- to post-treatment decline in PTSD and other trauma-related symptoms) in trauma-exposed children and adolescents (25–28). Additionally, several studies focusing specifically on youth involved in the legal system demonstrated the effectiveness of CBT-based interventions to assist with skills building, emotion management [as measured by the Difficulties in Emotion Regulation Scale (DERS)], violence and recidivism reduction, and improvement in functional outcomes such as program enrollment and employment obtainment (22, 29–31). Although the literature does not provide a clear distinction in outcomes for juvenile justice-involved youths who are children and adolescents vs. at a transitional age (emerging adults) supporting evidence suggests that addressing mental health, particularly deficits in dimensions of emotion regulation could be an effective method of intervention for the justice-involved emerging adults Roca serves.

An important link between trauma exposure and juvenile delinquency is impairment in emotion regulation. Emotion regulation is defined as the ability to correctly identify emotions and employ adaptive strategies to effectively address or cope with emotions (32, 33). Depression, substance use disorder, and PTSD can be understood as disorders of emotion dysregulation, with characteristic changes in the neurocircuitry of brain regions involved in threat-appraisal and fear learning. In early adolescence, there are key developmental changes happening in the brain, namely in the prefrontal cortex (PFC), which functions to support emotion regulation, impulse control, and personality expression (34). During the peri-pubertal (early preteen years), the PFC and parietal lobes begin protracted trimming of neuronal axons (a decrease of cortical gray matter) with a concomitant increase in myelination of axons (35). These neural changes are thought to underlie greater frontal cortex control over emotional behavior including impulsivity and poor decision-making (34). Trauma impacts brain development by contributing to impaired frontal cortex maturation and less top-down control, manifesting as impulsivity, inability to cope with intense emotion, poor decision-making, high novelty seeking behavior, and risk-taking in adolescents (36–41).

While maladaptive emotion regulation increases youths' risk for recidivism, adaptive emotion regulation may serve as a protective factor that could buffer against negative outcomes. Emotion dysregulation is correlated with externalizing problems including impulsivity, aggression, and substance use, which are linked to greater risk of arrest among youth (42, 43). In one study, emotion dysregulation, as reported by teachers, predicted the higher likelihood of arrest in adolescents (44). Conversely, greater self-control and self-regulation (e.g., impulse control, managing emotions) was correlated with reductions in delinquency and recidivism in youth (45, 46). In Docherty et al. (47), increased emotion regulation during incarceration was associated with lower risk for felony recidivism 1 year after youths' release from juvenile facilities. Moreover, adaptive emotion regulation is considered a resilience factor among violence-exposed youth (48). Thus, improving or strengthening emotion regulation skills may be an important strategy for reducing justice-involved youths' risk for (re)incarceration and improving their life outcomes. Taken together, addressing the underlying emotion dysregulation of justice-involved youth may be key for ensuring the safety and habilitation of youth from at-risk environments. Roca's CBT intervention model may provide an effective method to improve outcomes and reduce risk for violence and recidivism among justice-involved emerging adults, many of whom have experienced significant trauma.

The current study examined the effectiveness of Roca's CBT curriculum and approach on outcomes that may indicate risk for violence and recidivism: distress about life; criminal thinking, attitudes and behaviors; unhealthy relationship interactions; and drug and alcohol use (49, 50). Given prior evidence suggesting the effectiveness of CBT in improving emotion regulation, we examined the following research questions: (1) Is Roca's CBT intervention program associated with improvements in participants' emotion regulation? (2) Are improvements in emotion regulation associated with better behavioral and life outcomes, such as improvements in relationships, levels of life stress, and substance use, as well as decreases in criminal-related thinking, attitudes, and behavior? We hypothesized that the emerging adult men in Roca's program would improve in measures of emotion regulation, and that improved emotion regulation would be associated with improvements in participants' emotional and behavioral outcomes.

## Method

This was a data based, evaluation type project using data that was collected from all participants in the program as part of Roca's typical procedures. This study was IRB exempt because it included fully deidentified, retrospective data collected for clinical intervention purposes.

### Roca's intervention model

Data were collected as part of an academic-community partnership with Roca, a behavioral health intervention organization established in 1988. Roca has four programming sites in Massachusetts (Boston, Chelsea, Holyoke, and Springfield, MA) and one site in Baltimore, Maryland. The aim of the Roca intervention model is to reduce recidivism by: (1) creating safety and stability by fostering transformational relationships between youth workers/staff and participants, (2) teaching life-saving skills through education, job readiness, healthy habits, and parenting classes, (3) engaging participants in programming through relentless outreach by youth workers, (4) practicing skills via stage-based programming individualized for each participant, and (5) building strategic partnerships with institutions and systems (e.g., police and probation officers) (10).

### Roca's CBT curriculum

In 2016, Roca partnered with a Massachusetts General Hospital/Harvard Medical School expert in cognitive behavioral curriculum development for youth as an evidence-based practice to guide the construction of their CBT intervention. Utilizing a community-based participatory research framework and intervention mapping, the CBT curriculum was developed through iterative pilot testing and feasibility trials (51), and a training and coaching program was developed and implemented (52). The CBT skills curriculum was found to have good feasibility and acceptability among organizational staff and participants (51), and was effective in increasing youth program enrollment and job obtainment (53).

CBT was selected as the intervention modality in part because CBT-based programs used in correctional facilities, such as the Thinking for Change curriculum, are associated with reduced recidivism rates and improved emotional and behavioral outcomes in justice-involved youth (30, 54). CBT posits that maladaptive cognitions contribute to the maintenance of emotional distress and behavioral challenges. Based on the CBT model, maladaptive cognitions include general beliefs, or schemas, about the world, the self, and the future, giving rise to specific and automatic thoughts in particular situations. Strategies to change these maladaptive cognitions are associated with changes in emotional distress and behavioral challenges (55).

Roca's CBT curriculum concentrates on the following basic tenants: creating safety and stability; teaching life skills; practicing skills, rinse, and repeat; and engaging institutions and systems. Roca adapted the CBT model for youth to accommodate the reality that many of the youth that Roca wants to engage with and support are less likely to show up on their doorstep asking for support, secondary to their history of being sub-optimally and traumatically served by systems designed to help them. The Roca CBT adapted model applies a trauma-informed lens, adhering to trauma-informed principles of safety (psychological, physical), prevention of re-traumatization, collaboration, empowerment, mutuality, voice and choice, and respect for shared expertise (56). Meeting youth in an environment that feels safe to them (e.g., home, work, restaurant, school), Roca youth workers employ CBT based skills learning and invite youth to demonstrate their understanding of the skills. These CBT based interventions range from 10 to 60 min, once per month to once per year over a 3-year window, depending on the youth. The Roca CBT based trauma-informed model is about “meeting youth where they are”.

### Peer delivery of CBT

Roca uses a peer delivered CBT model in which masters level clinicians train paraprofessionals (e.g., youth workers who have similar backgrounds/lived experiences as participants, including involvement in gangs or the juvenile legal system) in Roca's CBT curriculum. Youth workers then deliver the CBT skills to emerging adult males in urban neighborhoods in Massachusetts and Maryland. Peer delivered CBT has been shown to be effective in improving relationships, treatment adherence and engagement, health outcomes, and anxiety and depression management (57, 58). Peer specialists meet participants where ever they feel most safe— literally meeting participants where they are (e.g., in participants' homes or on the street)—with the goal of increasing engagement in the intervention.

### Participants

Participants (*N* = 344) were emerging adult men aged 17–24 who engaged in the intervention model. The initial dataset included 1,896 participants; however, only participants who had at least three encounters (pre-, mid-, and post-CBT intervention) where they learned or engaged in CBT skills over a three-year window were included in analyses. Young men not included in this analysis did not meet the minimum engagement/participation criteria.

The average length of male emerging adult engagement with the Roca program is 2–4 years. The majority of the participants were of self-reported Hispanic/Latino ethnicity (55.8%) and were non-white (82.8%; Table 1). The majority did not finish high school (63.7%) and had not been employed within the past 6 months (68%) at intake. Furthermore, most participants were involved in the juvenile legal system at time of baseline interview (77.3%), had been involved in drug use or sale in the past (73.8%), and had been involved in street or gang activity (58.7%). Roca uses a risk assessment tool to determine whether participants are at the highest risk and to determine their individual dynamic and static risk factors (10). Young men were eligible to receive services if: (a) they were considered high-risk for (re)incarceration, gang involvement, substance use, or dropping out of high school and (b) if they are not ready, willing, or able to participate in traditional services or maintain employment (e.g., due to ongoing substance use, risk of harm to themselves or others, or noncompliance with organizations' requirements) (11). Young men were ineligible to receive services if they had a pending open charge in the adult legal system. Additionally, Roca assesses how ready participants are to engage in a change process. Participants who are assessed as low or medium risk are considered “ready to change” and are referred to more traditional youth development programs (10).

**Table 1:** Participant demographics at program intake (*N* = 344).

**Variable**	**%**
**Race**
American Indian or Alaskan Native	0.3
Asian	0.3
Bi/Multi Racial	14.2
Black or African American	34.9
Native Hawaiian or Other Pacific Islander	0.3
White	17.2
Other	29.4
**Ethnicity**
Hispanic/Latino	55.8
Non-Hispanic/Latino	42.4
**Education**
In High School	8.1
Dropped Out of High School – No GED	63.7
Dropped Out of High School – Has GED	10.8
Graduated High School	15.1
**Employment history (last 6 months)**
Has been employed	30.2
Has not been employed	68.0
**Is currently criminal justice system involved**
Yes	77.3
No	22.7
**Is drug involved (sale and/or use)**
Yes	73.8
No	26.2
**Is street/gang involved**
Yes	58.7
No	41.3

Variables with percentage totals non-equal to 100% reflect participants for whom we had missing data.

### Measures

The *Difficulties in Emotion Regulation Scale (DERS)* is a validated instrument measuring emotion dysregulation (59) including 36 items assessing six dimensions of emotion regulation (i.e., acceptance of emotional responses, emotional clarity, goal directed behavior, impulse control, emotional awareness, and emotion regulation strategies). These six dimensions are grouped into subscales which we used for analyses. Items were rated on a Likert scale from 1 (Almost never) to 5 (Almost always). Examples of items include “I am clear about my feelings,” “When I'm upset, I feel out of control,” and “When I'm upset, I have difficulty concentrating.” The DERS has good internal reliability, with each subscale having a Cronbach's α > 0.80 and the total scale having α = 0.93 (59). For the purposes of future reduced participant burden, a validated short form of the DERS (60) will be delivered moving forward, but for a more detailed analysis of the emotional regulation subscales the long form was included in the current analysis. We used the short form (DERS-SF) for all analyses except those that examined the subscales of the full DERS. A sum score of the DERS-SF items was created, with higher scores denoting less emotion regulation. For our analyses, we calculated a change score by subtracting the DERS-SF sum score from the first (pre) CBT skills session from the last (post) CBT skills session. Higher DERS-SF change indicates improved emotion regulation.

The *Life Distress Inventory (LDI)* is an 18-item rapid assessment inventory measuring self-reported distress in multiple areas of social life and functioning (e.g., intimate relationships, finances, and physical health) (61, 62). Participants rated the severity of distress they were feeling on a Likert scale from 1 (No distress) to 7 (The most distress ever felt) for each item. Higher scores indicated greater distress. The total LDI score has good reliability with a Cronbach's alpha of 0.89 (62).

The *Justification of Verbal and Coercive Tactics Scale* (63) (which is referred to as the *Relationship Interactions Questionnaire* in Roca's assessment battery and in the present manuscript) included 11 items assessing unhealthy relationship attitudes (e.g., “How justified is interfering with [your partner's] relationship with family members?” and “How justified is doing or saying something to spite [your partner]?”). Participants rated items on a Likert scale from 1 (Justified in MANY situations) to 5 (Not justified NO MATTER WHAT) with higher scores indicating healthier relationship interactions. The questionnaire has good internal consistency reliability (for males, Cronbach's α = 0.71 – 0.86, and for females Cronbach's α = 0.72 – 0.83) (63).

The 11-item *Drug/Alcohol Use Questionnaire* assessed number of days in the last month participants used alcohol and/or drugs, as well as the number of days they experienced alcohol and/or drug problems.

The *Criminal Thinking Scale (CTS)* is a brief 19-item self-report that assesses criminal thinking, attitudes, or behaviors (64). Using a 5-point Likert scale from 1 (Strongly disagree) to 5 (Strongly agree), example items from the CTS include “It is okay to commit crime in order to live the life you deserve” and “When not in control of a situation, you feel the need to exert power over others.” Roca administered three of the six subscales of the CTS: Entitlement (Cronbach's α = 0.78), Criminal Rationalization (Cronbach's α = 0.71), and Power Orientation (Cronbach's α = 0.81) (64). The sum score of the three CTS subscales was included in analyses. Higher scores on the CTS indicated more criminal thinking.

### Statistical analyses

Statistical analyses were completed using SPSS Statistics Version 24.0. The change in outcomes over time (pre-, mid-, post-) was assessed using repeated measures analyses of variance (ANOVA). The relationship between emotion regulation (as measured by the DERS-SF) and our outcome variables (e.g., level of life distress, quality of relationships, and drug/alcohol use) was analyzed using Pearson correlations and ANOVAs. The change in emotion regulation (DERS-SF) throughout the CBT skills program was the predictor variable. Given the multiple analyses performed to examine different aspects of life distress improvement, we performed a multiple-test correction on these specific analyses. Within the Life Distress Inventory (LDI), there are 18 items examining different components of life distress, and we performed 19 separate tests examining the LDI specific questions plus the sum score. Thus, for a simple Bonferroni correction, (0.05/19) a corrected alpha level for significance would be *p* = 0.0026. The outcome variables in our analyses included the total sum score and item-level variables from the LDI, Relationship Interactions Questionnaire sum score, Drug/Alcohol Use Questionnaire, and CTS sum score. To examine whether there might be unique relationships between our outcomes and different dimensions of emotion regulation (i.e., acceptance of emotional responses, emotional clarity, goal directed behavior, impulse control, emotional awareness, and emotion regulation strategies) we also completed Pearson correlations between the change scores of the six subscales of the DERS long form and our outcome variables.

## Results

### Change in outcomes over time

**Figure 1:**
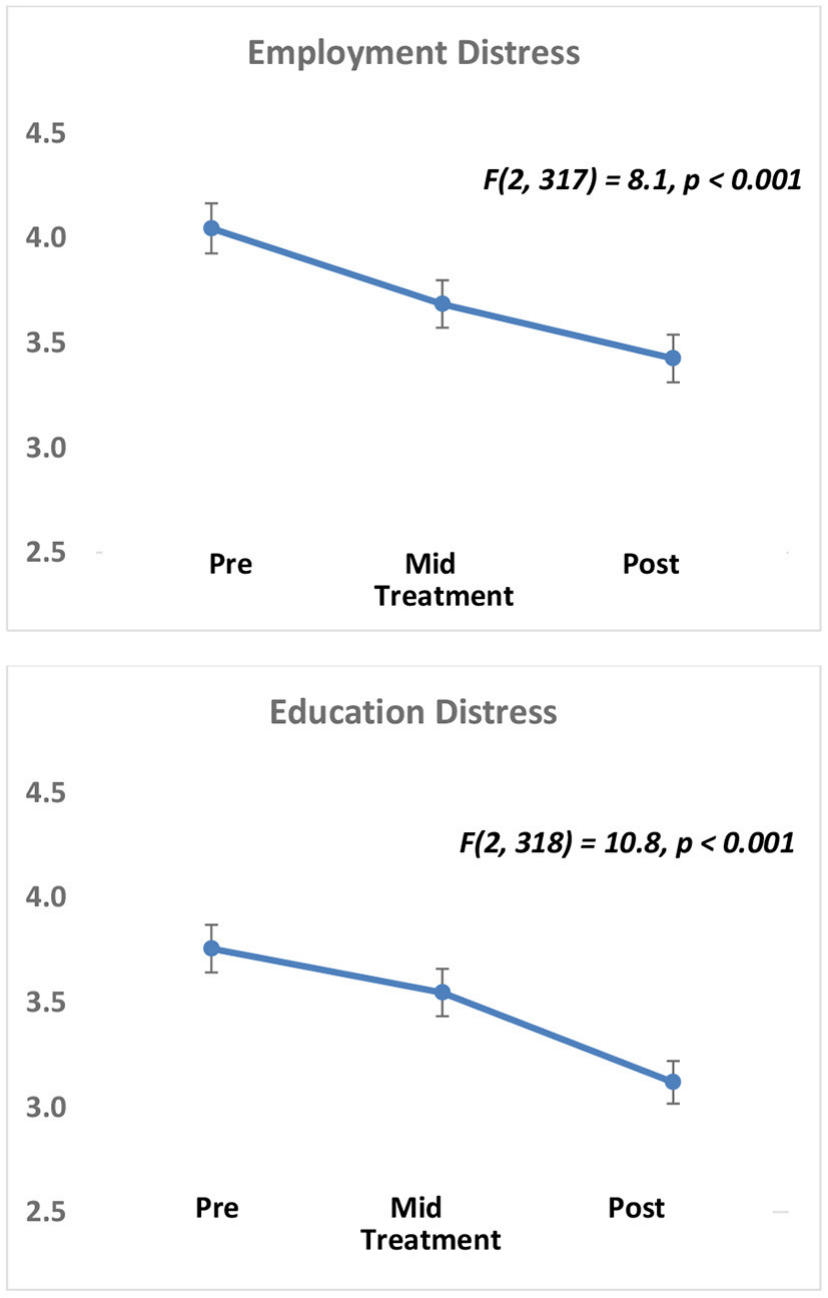
Improvement in employment and education-related distress across all Roca participants. Participants have significant reductions in Employment Distress (top) and Education Distress (bottom) across the three time points (Pre (intake), Mid (first skills-based appointment), and Post (most recent skills-based appointment) measured in this study. Distress is on a 1–5 scale (5 = most distress), plots show mean ± sem.

We initially examined whether psychological and quality of life measures improved across the course of treatment. Using rmANOVA with the full sample, we found that distress related to employment and education significantly improved over time. Specifically, pre-, mid-, and post-treatment employment distress (*F*_(2,319)_ = 8.5, *p* < 0.001, *η*_p_^2^ = 0.026) and education distress (*F*_(2,320)_ = 10.7, *p* < 0.001, *η*_p_^2^ = 0.032) showed robust and continuous improvement over time (Figure 1). Further, participants reported being less troubled or bothered by drug problems in the last 30 days (*F*_(2,286)_ = 304.3, *p* < 0.001, *η*_p_^2^ = 0.516) and felt that getting treatment for their drug problems was less important (*F*_(2,287)_ = 165.9, *p* < 0.001, *η*_p_^2^ = 0.366). However, there was a marginally significant increase in alcohol consumption over a 30-day period from pre-, mid-, to post-treatment (*F*_(2,295)_ = 3.1, *p* = 0.045, *η*_p_^2^ = 0.010).

### Is Roca's CBT intervention program associated with improvements in participants' emotion regulation?

A critical component of Roca that is distinct from most reintegration, education, job support, and youth development programs is the focus on mental health, behavior change and emotion regulation skills training. Using a paired samples *t*-test, we found that emotion regulation did not significantly improve across the entire sample (*p* = 0.256, as measured by the DERS-SF) from pre to post. These data suggested that not all participants were able to fully benefit from the structure and CBT skills, likely given this high-risk population is often living in ongoing at-risk situations. Due to this null finding, we were not able to test hypothesis two (i.e., are improvements in emotion regulation associated with better behavioral and life outcomes?) in the whole sample. Thus, the following exploratory analyses included all participants, but focused on comparing those whose emotion regulation improved (based on the DERS-SF) to those whose did not, to determine whether those with emotion regulation improvement were also more likely to have improvement across other quality of life and functional measures.

### Exploratory analyses – did those with improved emotion regulation also demonstrate better psychological and behavioral outcomes?

When we examined pre- to post- treatment change in the DERS-SF (calculated as post-DERS-SF minus pre-DERS-SF score), approximately half of the participants were found to have some improvement (positive score) in their DERS-SF over time. Specifically, based on the definition of greater emotion regulation total scores from DERS-SF at baseline to DERS-SF at the third assessment, *N* = 124 (46%) participants improved and *N* = 146 (54%) did not.

#### Life distress outcomes

In order to test whether improvements in emotion regulation associated with better life distress outcomes, we used ANOVA with DERS-SF category (improved vs. not-improved) predicting total LDI score or individual inventory item scores (19 tests).

**Figure 2:**
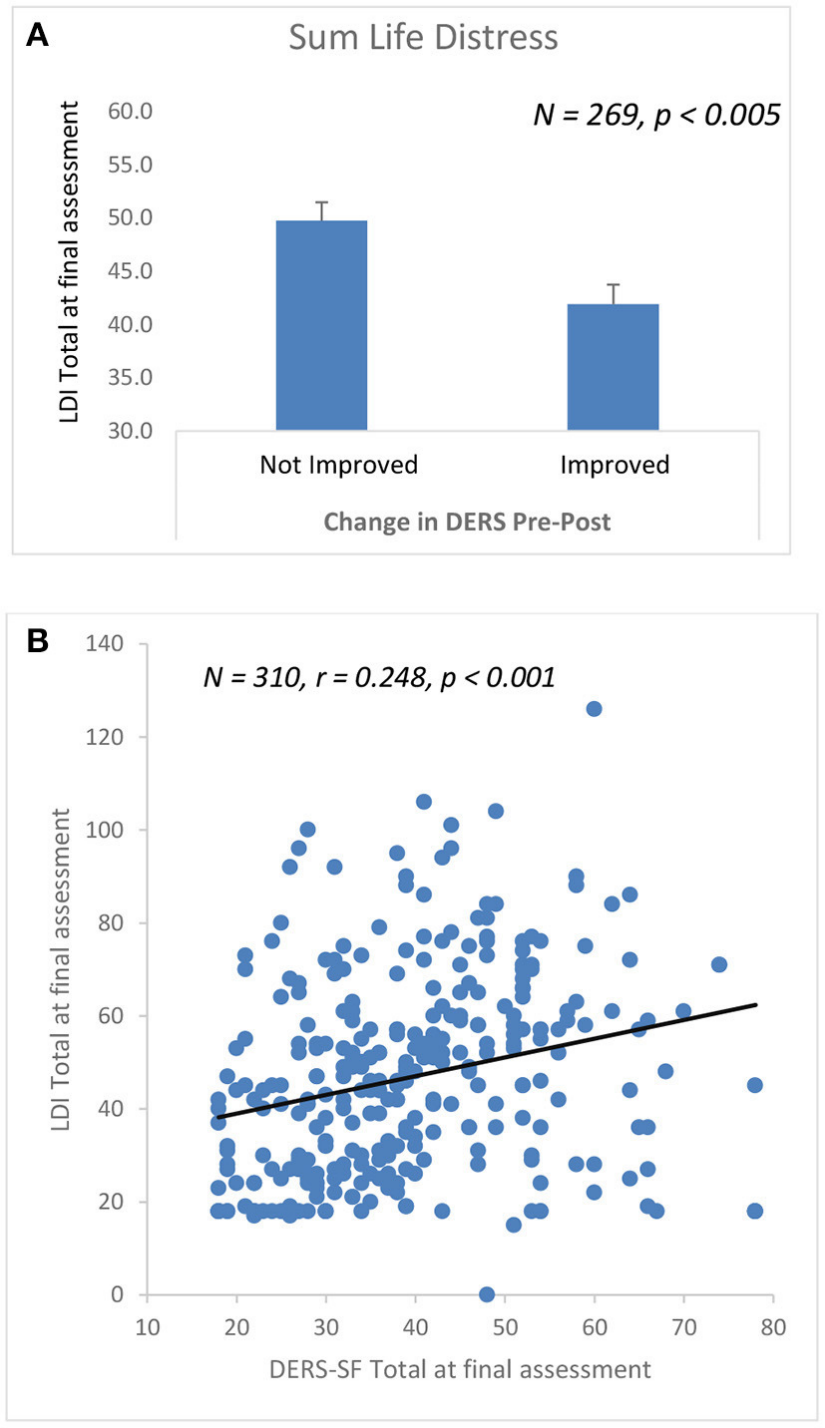
Improvement in emotion regulation associates with overall decreased life distress across Roca intervention. **(A)** Clients with greater improvement in emotion regulation over the course of ROCA CBT (higher levels of pre-post DERS-SF difference scores), also have significantly less Life Distress at last visit (*p* < 0.005). **(B)** Positive correlation between overall DERS-SFand sum LDI (e.g., better emotion regulation (lower DERS-SF), less life distress (lower LDI), (*r* = 0.248, *p* < 0.001, *N* = 310).

When we examined the role of emotion regulation improvement on overall life distress, we observed a robust difference in level of overall distress (sum LDI) in those with vs. without improved emotional regulation over time. Clients with greater improvement in emotion regulation over the course of Roca (higher levels of pre-post DERS-SF difference scores), also have significantly less life distress at last visit [*F*_(1,269)_ = 9.6, *p* = 0.002 *ω*^2^ = 0.031, 95% CI (0.001, 0.83); Figure 2A]. Furthermore, we observed a significant positive correlation between overall continuous emotion regulation measure (DERS-SF) and life distress (sum LDI) such that better emotion regulation is correlated with less life distress (*r* = 0.248, *p* < 0.001, *N* = 310) (Figure 2B).

**Figure 3:**
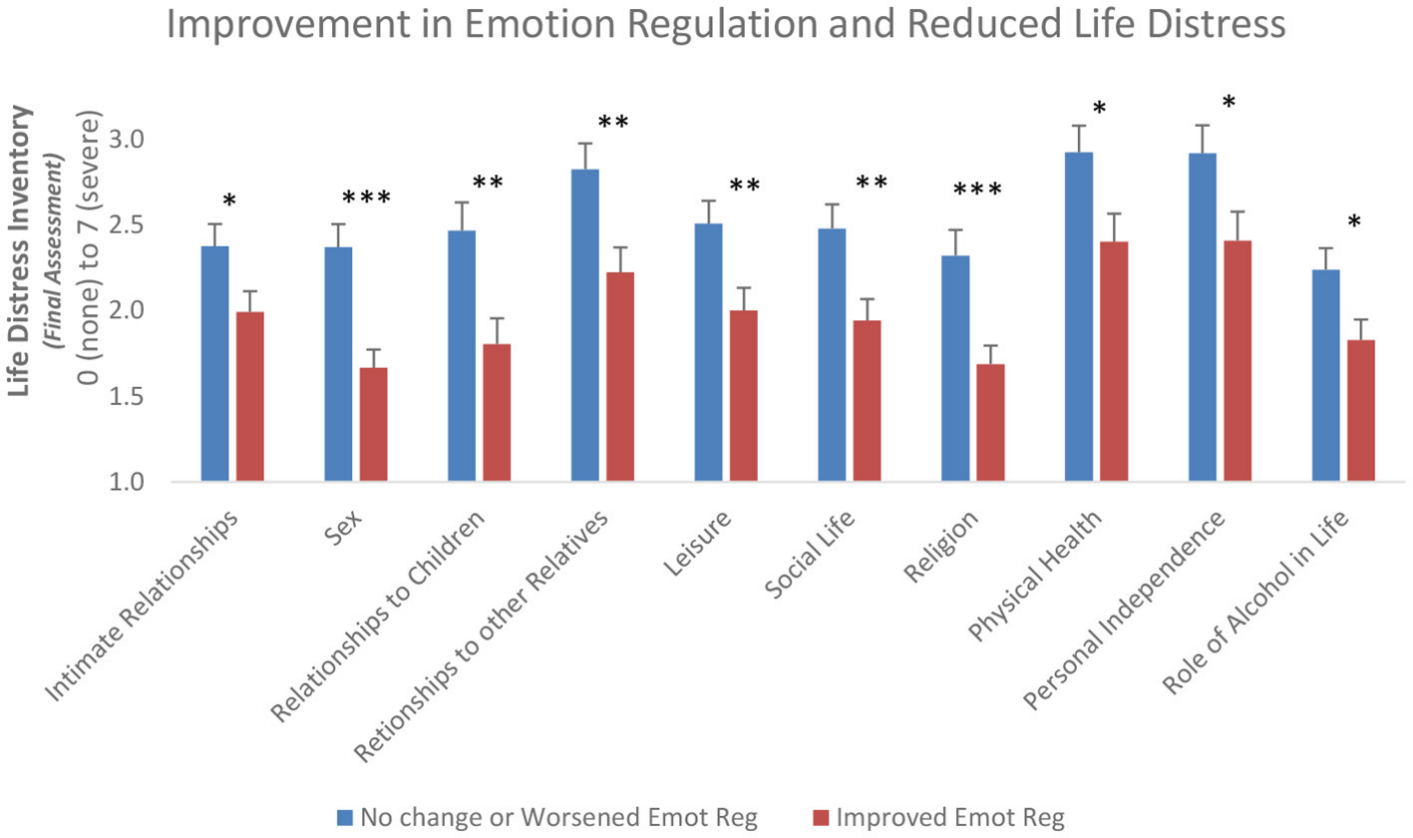
Improvement in Emotion Regulation associates with decreased Life Distress at final assessment. Participants whose Emotion Regulation improved over the course of the program also had less life distress in 10 of 18 areas at their final assessment. Participants who have overall improvement in Emotion Regulation (DERS-SF) (red bars) compared to those without improvement (blue bars) during the Roca CBT intervention demonstrate statistically significant lower life distress at the final assessment for 10 items on the Life Distress Inventory. Shown are mean scores + sem. *p < 0.05, **p < 0.01 and ***p < 0.001.

Further, we found that, compared to participants who did not demonstrate improved emotion regulation from pre-to-post CBT, participants who did demonstrate improved emotion regulation had significantly lower ratings across 10 of the 18 areas of life distress at the follow-up timepoint. LDI individual items that were found to be nominally significant as a function of DERS-SF outcome included distress related to intimate relationships [*F*_(1,266)_ = 4.5, *p* = 0.03, *ω*^2^ = 0.013, 95% CI (−0.004, 0.055)], sexual behavior [*F*_(1,268)_ = 15.9, *p* < 0.001, *ω*^2^ = 0.052, 95% CI (0.011, 0.113)], relationship to children [*F*_(1,261)_ = 8.7, *p* = 0.003, *ω*^2^ = 0.029, 95% CI (0.00, 0.081)] and relationships to other relatives [*F*_(1,263)_ = 8.0, *p* = 0.005, *ω*^2^ = 0.026, 95% CI (−0.001, 0.077)], recreation/leisure [*F*_(1,265)_ = 7.1, *p* = 0.008, *ω*^2^ = 0.022, 95% CI (−0.002, 0.071)], social life [*F*_(1,264)_ = 7.7, *p* = 0.006, *ω*^2^ = 0.025, 95% CI (−0.001, 0.075)], religion [*F*_(1,267)_ = 11.0, *p* = 0.001, *ω*^2^ = 0.036, 95% CI (0.003, 0.091)], physical health [*F*_(1,267)_ = 5.3, *p* = 0.02, *ω*^2^ = 0.016, 95% CI (−0.004, 0.060)], personal independence [*F*_(1,267)_ = 4.6, *p* = 0.03, *ω*^2^ = 0.013, 95% CI (−0.004, 0.056)], and with regards to the role of alcohol in their life [*F*_(1,267)_ = 5.5, *p* = 0.02, *ω*^2^ = 0.017, 95% CI (−0.003, 0.061); Figure 3]. Given the multiple analyses performed to examine different aspects of life distress improvement, we performed a Bonferroni multiple-test correction on these analyses, with a corrected alpha level of *p* = 0.0026. With this correction for multiple testing on the LDI individual items, sexual behavior and religion would survive the analyses.

In addition to the categorical analyses of DERS-SF outcome, we examined the bivariate correlation between continuous variables of improvement on the DERS-SF (DERS-SF change score) related to the above items on the Life Distress Inventory for all participants. In addition to an overall significant correlation with total LDI (*r* = −0.128, *p* < 0.05), we found significant correlations between improvement on the DERS-SF and LDI items of sexual behavior (*r* = −0.189, *p* < 0.01), relationship to children (*r* = −0.165, *p* < 0.01), social life (*r* = −0.202, *p* < 0.001), recreation/leisure (*r* = −0.185, p < 0.01) and religion (*r* = −0.223, *p* < 0.001). Following a correction for multiple testing (Bonferroni adjusted alpha = 0.0026), sex, recreation/leisure, social life, and religion would survive the continuous analyses. Together, these data suggest that the effect of the CBT intervention on improving emotion regulation may result in improvements in several of the life distress items that were measured.

#### Relationship interactions, drug/alcohol consumption, and criminal thinking

In order to test whether improvements in emotion regulation associated with better relationship, criminal thinking, and drug/alcohol outcomes, we used ANOVA with DERS-SF category (improved vs. not-improved) predicting total RI and CTS sum score or individual drug/alcohol item scores at the final timepoint. When we examined the role of emotion regulation improvement on relationship interactions and criminal thinking, we did not observe a difference in RI sum score (*p* = 0.107) or CTS sum score (*p* = 0.078) in those with vs. without improved emotional regulation over time. Similarly, we did not observe a significant difference in any of the drug/alcohol consumption items between those with vs. without improved emotional regulation over time. In addition to the categorical analyses of relationship interactions, drug/alcohol consumption, and criminal thinking outcomes, we examined the bivariate correlation with continuous variables of improvement on the DERS-SF and found that emotion regulation improvement was not significantly correlated with any of these outcomes.

### Exploratory analyses - association between emotion regulation subscales and behavioral outcomes

**Figure 4:**
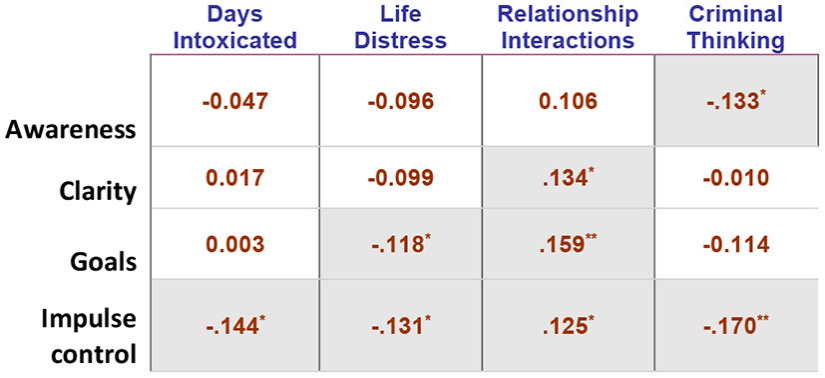
Correlation table of change in emotion regulation and outcome indicators. Table shows Pearson ‘*r*' correlation between change (pre-to-post) score on 4 of the 6 DERS subscales and several outcome indicators at final assessment. Gray cells are significant, * <0.05, ** <0.01. For directionality of associations, note that: higher score on RI = healthier relationship interactions; higher score on LDI = more distress; higher score on DERS = less emotion regulation; higher score on CTS = more criminal thinking; higher DERS change = improved emotion regulation.

We also observed several correlations between change in emotion regulation (DERS) subscales (e.g., acceptance of emotional responses, emotional clarity, goal directed behavior, impulse control, emotional awareness, and emotion regulation strategies) and other outcome indicators (Figure 4). While we did not observe a significant overall improvement in emotion regulation from pre to post, there was a significant improvement in the emotional awareness subscale of the DERS from pre to post [*t*_(310)_ = −3.08, *p* = 0.002, Cohen's *d* = 0.174, 95% CI (0.062, 0.286)]. Further, we found that improved emotional awareness correlated with less criminal thinking, more emotional clarity was associated with improved relationship functioning, and less difficulty in engaging in goal directed behavior was associated with less life distress and improved relationship interactions. Notably, the DERS subscale related to improved impulse control was associated with the most positive outcome including decreased life distress, improved relationship functioning, decreased criminal thinking, and decreased alcohol use (*p*'s <0.05, *r*'s = 0.125 to 0.159; Figure 4).

## Discussion

Roca's trauma-informed CBT targets improving emotion and cognitive regulation in challenging circumstances. In line with earlier work using participant data from Roca's intervention model (53), we found that at-risk emerging adults who participated in the CBT curriculum experienced less distress about employment and education over time. These findings likely relate to the structure, education, and job placement components of the supportive reintegration programming for justice involved youth. Moreover, the results of the present study provide supporting evidence that CBT skills contribute to improvements in emotional awareness, one aspect of emotion regulation, and that those with improved emotion regulation also had significantly less distress about multiple life areas. We also found that improvement in emotional clarity, goal directed behavior, impulse control, and emotional awareness correlated with greater improvements in outcome measures, including lower levels of distress in several areas of life, less criminal thinking, healthier relationship interactions, and reduced substance and alcohol use. Although this sample as a whole did not experience statistically significant improvement in emotion regulation, these above results provide preliminary evidence that one effective way to achieve the goals of habilitation and skills building programs is to address the underlying emotion dysregulation that stems from trauma and contributes to violence perpetration.

Noteworthy among our findings is that despite positive associations between the subscales of emotion regulation and improved outcomes, emotion regulation across all domains did not improve over time for the overall group. The limited improvements in emotion regulation after receiving CBT skills may be explained by several factors. First, due to the naturalistic nature of this study, all individuals received CBT intervention but not all individuals received the same amount of CBT intervention. Further, prior literature has found that certain types of CBT are more effective than others with specific CBT treatment elements moderating outcome effectiveness. For example, a meta-analysis on CBT effectiveness found that CBT programs that included anger control and interpersonal problem-solving skills were more effective at reducing recidivism while those that included victim impact or behavior modification skills were less effective (30).

Furthermore, behavior and emotion change take time to develop, and emotion regulation skills may take longer to establish in persons who have experienced profound, often developmental, trauma that Roca serves (5, 42, 44, 46). Key tenets in Roca's programming are building trust and safety with individuals but this too takes time to foster and is often a pre-requisite for affective and behavioral change (65). It is promising that even though the DERS overall was not significantly associated with improved outcomes in the entire sample, certain DERS subscales were associated with lower life stress in areas such as social life, religion, and sexual behaviors. Among the DERS subscales that were correlated with improved life outcomes were the emotion awareness and emotion clarity subscales. It is possible that the first step in changing one's actions in relation to one's emotions is correctly identifying and labeling the emotions one is feeling. Thus, individuals' may be experiencing affective changes in association with the CBT intervention, but the DERS isn't a sensitive enough tool to measure small, incremental improvements. Using a different measure or re-administering the DERS after individuals' have received more CBT sessions may yield different results. Additionally, it would be useful to collect collateral (e.g., youth worker or peer reports) in addition to self-report measures.

Moreover, the characteristics of CBT recipients has been shown to influence outcomes. Persons served by Roca have experienced significant trauma and have challenging life circumstances; this could provide an explanation for differences in effectiveness. For example, consistent with the “risk-principle” (66) those with disproportionately challenging circumstances and who have significant trauma experiences may have had higher susceptibility at the start of program and may have benefitted more from treatment. Similarly, differences in individuals' trauma exposure or mental health status at the start could determine who benefits more from the CBT. These observations suggest that expanded and culturally sensitive engagement strategies may be critical for those at higher risk.

Furthermore, studies have found that the amount and implementation of the CBT moderated the effectiveness on CBT (30, 31). Variability in the amount/frequency of exposure to CBT could explain the limited improvements in emotion regulation in some individuals and should be examined in the future. Importantly, this curriculum was designed to be flexibly taught whenever needed, in brief segments and in formal or informal settings. Our results demonstrate improvement in emotion regulation and outcomes in some individuals, suggesting that the method of curriculum delivery may be effective with certain skills but not others, or more effective with some clients than others. Expanding our understanding of the most effective aspects of interventions, and the most effective way to pair specific interventions with individuals, will further the long-term success of such programs.

The results of this study provide initial evidence that a community-based, CBT intervention for justice-involved emerging adult men can positively impact affective, behavioral, and life outcomes, including improved emotion regulation, decreased criminal thinking, less life distress, improved relationships, and decreased alcohol use. These findings are consistent with previous meta-analyses and reviews that found that CBT-based programs for individuals who have become entangled with the legal system, including youth who have become entangled or more susceptible to entanglement with the legal system, were associated with improvements in outcomes, including small but significant reductions in recidivism and externalizing behaviors (67–69). Additionally, Trauma Affect Regulation: Guide for Education and Therapy (TARGET) (70), a trauma-informed cognitive-behavioral intervention for adolescents, was associated with greater improvements in depression and participant-rated optimism and hope compared to treatment as usual in a group of incarcerated youth (71). Therefore, CBT-based interventions, especially those that are trauma-informed, demonstrate positive associations with life and behavioral outcomes in justice-involved populations.

## Limitations and future directions

The present findings should be considered in light of several limitations. First, we did not consider environmental and situational contexts of the individuals' lives outside of their qualifying risk factors, nor other sources of institutional support they may be receiving. These factors, such as whether they have a support system outside of Roca, could potentially influence the effectiveness of the curriculum. For example, healthy interactions with family is one avenue that has been found to positively impact anger regulation in youth exposed to violence (72). Second, Given Roca's intervention model of meeting individuals where they are at (both practically and figuratively), a large proportion of our initial sample received less than three CBT skills sessions. An important aspect of building trust and safety is allowing youth to decide when they are ready to engage in a new intervention, and where and how they would like that engagement to happen. However, in addition to greatly limiting our sample size and thus reducing our statistical power, inconsistent or limited engagement in the intervention may have influenced the overall effectiveness of the CBT intervention in improving emotion regulation. Third, we were not able to include “dosing” of amount of exposure to CBT that individuals had in this analysis as described above, nor were we able to have a true “control” group, though future studies may be able to utilize a wait-list control. Fourth, based on the naturalistic nature of this study, we did not have information about youth worker assignments (e.g., which youth workers engaged with which individuals and how often). Moreover, it is possible that differences in the training and/or supervision of youth workers could have impacted the effectiveness of the CBT intervention, although previous work has demonstrated the fidelity of the intervention model (51, 53). Lastly, given that the study sample included emerging adult males aged 17–24, the study results may have limited generalizability to other populations, such as females and other age groups involved in the juvenile legal system. Furthermore, data for this study was collected as part of one organization's routine programming. An important next step would be to see if the program's CBT curriculum is generalizable to other organizations or geographical locations.

Recognizing these limitations, the study findings offer a number of meaningful directions for future research. First, examining contextual factors as potential moderators of the effect of Roca's CBT programming on outcomes could help us understand why some individuals improved in their emotion regulation and others did not. Mediation analyses would also further allow us to examine whether the emotion regulation changes are causal in the observed overall improvements in outcomes. Furthermore, future inclusion of additional objective re-incarceration related outcome measures would help provide a stronger case for CBT's effects on recidivism.

The present study examines the effect of CBT on behaviors and attitudes that may confer susceptibility for violence and recidivism. These findings suggest that CBT skills are associated with positive improvements in emotion regulation, such as an improvement in emotional awareness, which in turn could potentially impact life outcomes such as rates of violence and recidivism in justice-involved youth. Despite the limitations, these findings contribute to our understanding of the effectiveness of CBT programs for youth who have experienced trauma, have challenging life circumstances, have become entangled or who may be more susceptible to becoming entangled with the legal system. This research can be used to inform future efforts to improve the lives of justice-involved emerging adults.

## Data availability statement

The raw data supporting the conclusions of this article will be made available by the authors, without undue reservation.

## Ethics statement

The studies involving human participants were reviewed and approved by Mass General Brigham Institutional Review Board. Written informed consent from the participants' legal guardian/next of kin was not required to participate in this study in accordance with the national legislation and the institutional requirements.

## Author contributions

KR and AM-C contributed to funding support and conception and design of the study. AC, LE, MB, and SK collected data from Roca, organized intervention, and organized the database. KR and ND performed the statistical analysis. ND, KF, AM-C, and KR wrote the first drafts of the manuscript. AC, LE, MB, SK, SY, and LM wrote sections of the manuscript. All authors contributed to manuscript revision, read, and approved the submitted version.

## Funding

This work was completed as part of a community-academic partnership between Roca and McLean Hospital, funded by a grant from the Bank of America Foundation, as well as the Trauma Initiative at McLean Hospital, and the McLean Frazier Institute.

## Conflict of interest

Authors AC, LE, SK, and MB were employed by Roca, Inc. Author AM-C worked as a subject matter expert through a federally funded SMART Grant in partnership with the Multnomah County Department of Community Justice (DCJ) and received payment for her work. Author KR has received consulting income from Alkermes and Takeda, research support from NIH, Genomind, and Brainsway, and he is on scientific advisory boards for Janssen and Verily, none of which is related to the present work. The remaining authors declare that the research was conducted in the absence of any commercial or financial relationships that could be construed as a potential conflict of interest.

## Publisher's note

All claims expressed in this article are solely those of the authors and do not necessarily represent those of their affiliated organizations, or those of the publisher, the editors and the reviewers. Any product that may be evaluated in this article, or claim that may be made by its manufacturer, is not guaranteed or endorsed by the publisher.
